# Blood-Brain Barrier Breakdown and Blood-Brain Communication in Neurological and Psychiatric Diseases

**DOI:** 10.1155/2011/431470

**Published:** 2011-07-06

**Authors:** Alon Friedman, Daniela Kaufer

**Affiliations:** ^1^Department of Physiology and Biomedical Engineering, Faculty of Health Sciences and Zlotowski Center for Neuroscience, Ben-Gurion University of the Negev, Beer-Sheva 84105, Israel; ^2^Department of Integrative Biology and Helen Wills Neuroscience Institute, University of California, Berkeley, CA 94720-3140, USA

More than a century ago, Paul Ehrlich demonstrated in a set of dye experiments the lack of permeability of intracerebral vessels to albumin-binding dyes and therefore postulated a barrier between blood and neuronal tissue. Indeed, transport across the blood-brain barrier (BBB) is tightly regulated by at least four different cells that comprise the brain microvasculature: the endothelial cell and highly specific tight junctions between them, the pericytes which share with the endothelial cells a common capillary basement membrane, the astrocytic foot processes which cover the capillaries, and nerve endings which innervate the vessels. Importantly, dysfunction of the BBB occurs during numerous common neurological diseases, including stroke, epilepsy, trauma, tumors, and infectious and degenerative diseases. While it has been long recognized that BBB dysfunction is associated with brain diseases, only recently it has been suggested to play a role in the pathogenesis of neuronal networks dysfunction and degeneration. In this special issue clinical and experimental evidence for the involvement of BBB dysfunction in the pathogenesis of seizures and epilepsy (N. Marchi et al. “*the etiological role of blood-brain barrier dysfunction in seizure disorders*” and L. M. Gibson et al. in “*Occult cerebrovascular disease and lateonset epilepsy: could loss of neurovascular unit integrity be a viable model?*”), posttraumatic epilepsy (O. Tomkins et al. in “*Blood-brain barrier breakdown following traumatic brain injury: a possible role in posttraumatic epilepsy*”), Alzheimer's diseases (V. C. Anderson et al. in “*The blood-brain barrier and microvascular water exchange in Alzheimer's disease*”), and psychiatric disorders (Y. Serlin et al. in *“Vascular pathology and blood-brain barrier disruption in cognitive and psychiatric complications of type 2 diabetes mellitus”*) is given. Experimental evidence points to the mechanisms involved, which most importantly seems to include astroglial activation and disturbance of the extracellular milieu, specifically altered homeostasis of water and electrolytes (V. C. Anderson et al. in *“The blood-brain barrier and microvascular water exchange in Alzheimer's disease”*). In addition, immune response and inflammation seems to have closed bidirectional interactions with disturbed BBB permeability (H. B. Stolp et al. in *“Effects of neonatal systemic inflammation on blood-brain barrier permeability and behaviour in juvenile and adult rats,”* A. S. Haqqani and D. B. Stanimirovic in *“Intercellular interactomics of human brain endothelial cells and Th17 lymphocytes: a novel strategy for identifying therapeutic targets of CNS inflammation,”* and A. R. Friedman et al. in *“Elucidating the complex interactions between stress and epileptogenic pathways”*). 

The accumulating experimental evidence for BBB involvement in the pathogenesis and progression of these common neurological diseases raises important unresolved questions of how similar vascular dysfunction can lead to wide range of neurological symptoms and signs. While the answers to these key questions are not yet known, papers in this special issue tackle some of the potential variables including the localization, extent, and duration of BBB dysfunction (Y. Serlin et al. in *“Vascular pathology and blood-brain barrier disruption in cognitive and psychiatric complications of type 2 diabetes mellitus”*), the time point during development (H. B. Stolp et al. in *“Effects of neonatal systemic inflammation on blood-brain barrier permeability and behaviour in juvenile and adult rats”*) and aging (L. M. Gibson et al. in *“Occult cerebrovascular disease and late-onset epilepsy: could loss of neurovascular unit integrity be a viable model?”*) in which disturbance occurs, and the interaction with confounding factors such as stress in early life or in adulthood (A. R. Friedman et al. in *“Elucidating the complex interactions between stress and epileptogenic pathways”*). These open questions raise the need for the development of new methods for the study of BBB dysfunction exvivo—as described by R. Kovács and colleagues (in *“Slice cultures as a model to study neurovascular coupling and blood brain barrier in vitro”*). Furthermore, it becomes clear that methods for the quantitative and reliable evaluation of BBB permeability are lacking. In this respect, new imaging approaches in experimental animals (D. Jorks et al. in *“A novel algorithm for the assessment of blood-brain barrier permeability suggests that brain topical application of endothelin-1 does not cause early opening of the barrier in rats”*) and in humans (O. Tomkins et al. in *“Blood-brain barrier breakdown following traumatic brain injury: a possible role in posttraumatic epilepsy”* and V. C. Anderson et al. in *“The blood-brain barrier and microvascular water exchange in Alzheimer's disease”*) are presented as part of the ongoing effort to allow the diagnosis, followup, and evaluation of the integrity of the neurovascular unit and BBB functions. Finally, the new concepts and mechanisms described recently in the literature highlight the neurovascular unit including specifically brain vessels and immune system as new therapeutic targets for the prevention and treatment of neurological diseases. Novel approaches for the identification of new targets based on complex genomic, proteomic, and interactomics tools are presented by A. S. Haqqani and D. B. Stanimirovic (in *“Intercellular interactomics of human brain endothelial cells and Th17 lymphocytes: a novel strategy for identifying therapeutic targets of CNS inflammation”*).

*Alon Friedman* *Daniela Kaufer*

